# Dual-color plasmonic enzyme-linked immunosorbent assay based on enzyme-mediated etching of Au nanoparticles

**DOI:** 10.1038/srep32755

**Published:** 2016-09-07

**Authors:** Longhua Guo, Shaohua Xu, Xiaoming Ma, Bin Qiu, Zhenyu Lin, Guonan Chen

**Affiliations:** 1 Institute of Nanomedicine and Nanobiosensing; Ministry of Education Key Laboratory of Analysis and Detection Technology for Food Safety; College of Chemistry, Fuzhou University, Fuzhou, 350116, China

## Abstract

Colorimetric enzyme-linked immunosorbent assay utilizing 3′-3-5′-5-tetramethylbenzidine(TMB) as the chromogenic substrate has been widely used in the hospital for the detection of all kinds of disease biomarkers. Herein, we demonstrate a strategy to change this single-color display into dual-color responses to improve the accuracy of visual inspection. Our investigation firstly reveals that oxidation state of 3′-3-5′-5-tetramethylbenzidine (TMB^2+^) can quantitatively etch gold nanoparticles. Therefore, the incorporation of gold nanoparticles into a commercial TMB-based ELISA kit could generate dual-color responses: the solution color varied gradually from wine red (absorption peak located at ~530 nm) to colorless, and then from colorless to yellow (absorption peak located at ~450 nm) with the increase amount of targets. These dual-color responses effectively improved the sensitivity as well as the accuracy of visual inspection. For example, the proposed dual-color plasmonic ELISA is demonstrated for the detection of prostate-specific antigen (PSA) in human serum with a visual limit of detection (LOD) as low as 0.0093 ng/mL.

Colorimetric enzyme-linked immunosorbent assay (ELISA) is one of the most popular techniques for clinical diagnosis of diverse diseases^1^,^2^,^3^. Most of commercially available colorimetric ELISAs employ horseradish peroxidase (HRP) as the enzyme label and 3,3′,5,5′-tetramethylbenzidine (TMB) as the chromogenic substrate^4^,^5^. In the presence of targets, HRP catalyzes the oxidation of TMB (colorless) into TMB^2+^ (yellow)^6^, and the concentration of targets is quantified on the basis of the optical density of the yellow solution^7^,^8^. Naked-eye inspection of the targets is inaccurate because human eyes are insensitive to optical density variations of the same color. Normally, the optical density of colorimetric ELISA is detected with a sophisticated readout (e.g. an automatic microplate reader) in the hospital^9^,^10^. However, in some cases, the use of a costly and bulky readout is not applicable. For example, portable and affordable devices are greatly welcome for in-home personal healthcare^11^,^12^,^13^,^14^,^15^,^16^. Therefore, the development of colorimetric ELISA with improved accuracy for naked-eye inspection could be greatly desired.

In the past two decades, the unique optical properties of noble metal nanoparticles have attracted intense attention to both the academic and industrial communities^17^,^18^,^19^,^20^,^21^,^22^. For instance, Stevens and coworkers incorporated the optical responses of noble metal nanoparticles into conventional ELISA to develop a novel immunoassay strategy called plasmonic ELISA^14^,^23^,^24^,^25^. This new ELISA method was based on enzyme-controlled growth of gold nanoparticles (AuNPs). In the absence of analyte, the reduction of gold ions was guided to form quasi-spherical, non-aggregated gold nanoparticles, and the solution color was red; in the presence of analyte, the reduction of gold ions was guided to form an ill-defined morphology comprising aggregated nanoparticles, and the solution color was blue. The color transformation from red to blue was easily distinguishable with the naked eyes^24^. Thus the proposed plasmonic ELISA showed an ultralow limit of detection for prostate specific antigen (PSA) and HIV-1 capsid antigen p24 (1 × 10^−18^ g/mL). Jiang and coworkers^26^ developed a strategy to conduct plasmonic ELISA based on click chemistry induced formation of AuNP aggregates. This work can well accommodate conventional immunoassays by employing alkaline phosphatase (ALP) as the enzyme label. Note that all above mentioned plasmonic ELISA methods present color variations from red to blue in response with different concentrations of targets. The red and blue colors are easily to distinguish with the naked eyes; however, the discrimination of colors in-between red and blue are still a big challenge with the naked eyes. In addition, color variations between red and blue result in continuous changes of the absorption spectral peaks, which is inconvenient to be monitored with a microplate reader for the quantification of the analytes.

In this work, we demonstrate another alternative to conduct plasmonic ELISA based on enzyme mediated etching of gold nanoparticles. Our approach displays two distinct colors in responses of different concentrations of analytes, namely, the solution gradually changed from wine red to colorless, and then from colorless to yellow with an increase amount of analytes. Therefore, during the whole detection process, the absorption spectra of the solution only shows two peaks; one located at ~530 nm, which corresponds to the wine red solution; the other located at ~450 nm, which corresponds to the yellow solution. These two distinct absorption peaks are easily to be detected with the microplate reader. Comparing with the conventional ELISA, this dual color plasmonic ELISA can effectively improve the accuracy of visual inspection.

## Results and Discussion

### Principle of the dual-color ELISA

**Figure 1:**
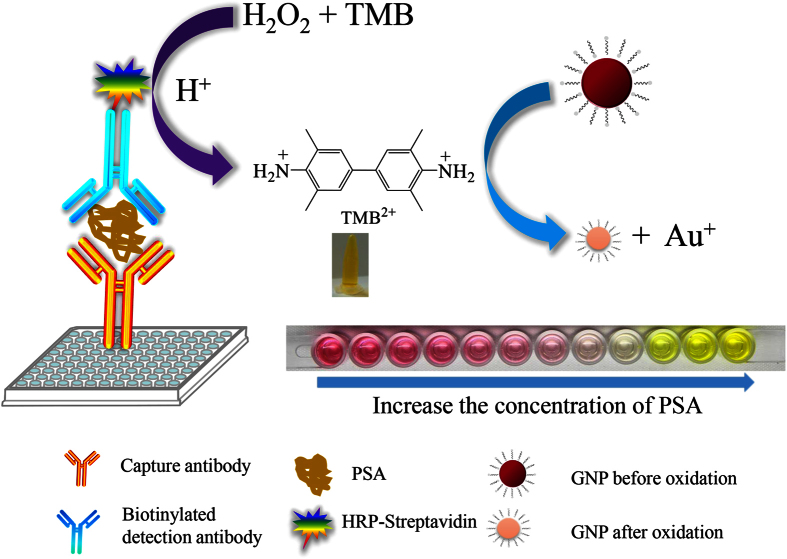
Principle of the dual-color ELISA for visual detection of protein.

Figure 1 depicts the principle of the dual-color ELISA. Generally, our detection scheme can be divided into two steps. The first step was a standard ELISA process, which was directly adopted from a commercial available ELISA Kit. The target molecule (antigen) was immobilized on the microplate via antibody-antigen interaction; then a second antibody conjugated with HRP was immobilized onto the antigen. After the addition of enzymatic reaction substrate containing peroxide and TMB, HRP would catalyze the oxidation of the colorless TMB into blue TMB^+^. The blue TMB^+^ would turn into yellow TMB^2+^ after the addition of a stop solution containing hydrochloric acid. The concentration of TMB^2+^ was proportional to the concentration of antigen in the assay samples, which was the basis of the commercial ELISA for quantitative determination of the concentration of proteins. The second step was the colorimetric reaction process, and this step was the main difference between our method and the conventional ELISA. An appropriate amount of CTAB-capped AuNPs was added to the yellow solution containing TMB^2+^ which was produced in the first step. The solution color turned into red after the addition of AuNPs (Fig. 1). The optical density of the red solution decreased with the increase of the analyte concentration, and the solution was then turned into colorless when further increasing the analyte concentration. The colorless solution indicated that there was neither AuNPs nor TMB^2+^ in the solution. Higher concentration of analytes would turn the solution into yellow color. This yellow solution indicated the presence of excess TMB^2+^.

### Proof and mechanism of TMB^2+^ induced etching of AuNPs

**Figure 2:**
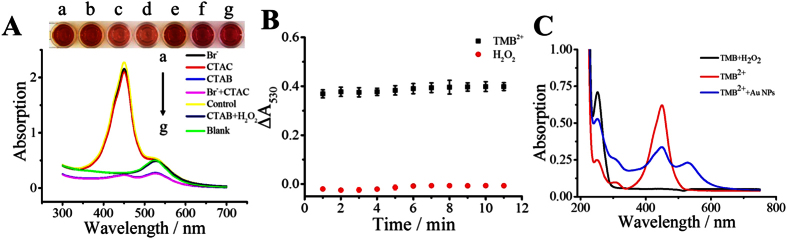
The mechanism of TMB^2+^ etching AuNPs. (**A**) The absorption spectrum of AuNPs (100 μL, tween-20 protected) in the presence of different additives. (a) 50 μL 0.1 M Br^−^ and 50 uL 0.2155 mM TMB^2+^; (b) 50 μL 0.1 M CTAC and 50 uL 0.2155 mM TMB^2+^; (c) 50 μL 0.1 M CTAB and 50 uL 0.2155 mM TMB^2+^; (d) 25 μL 0.2 M Br^−^ and 25 μL 0.2 M CTAC and 50 uL 0.2155 mM TMB^2+^; (e) 50 uL H_2_O and 50 uL 0.2155 mM TMB^2+^; (f) 50 uL 0.1 M CTAB and 50 uL 1.5 mM H_2_O_2_; (g) 100 uL H_2_O, insert was the corresponding colorimetric picture. (**B**) The absorption decrease value of AuNPs added with TMB^2+^ and H_2_O_2_ at different reaction time. (**C**) The UV-vis spectrum of TMB liquid substrate system used in this work, 0.2104 mM TMB^2+^ produced by the TMB system and 100 uL AuNPs with 100 uL 0.2104 mM TMB^2+^, all these three mixtures were diluted 10 times.

To show the experimental evidence of TMB^2+^ induced etching of AuNPs, control studies were performed to investigate the effect of TMB^2+^, Br^−^, and CTA^+^ on etching process of AuNPs. As shown in Fig. 2A, the absorption value (450 or 530 nm) seldom changed when only Br^−^ or CTA^+^ existed (sample a and b); they showed an orange red color (the tertiary color of red and yellow) which was the same as the control (only AuNPs and TMB^2+^ existed, sample e). On the contrary, when CTAB or CTA^+^ and Br^−^ existed (sample c and d), a sharp decrease appeared and the color of the mixture correspondingly changed from red to pink. These results demonstrate that (1) neither the mixture of TMB^2+^ and Br^−^ nor the mixture of TMB^2+^ and CTAC can induce significant etching of AuNPs; and (2) either the presence of the mixture of TMB^2+^ and CTAB or the mixture of TMB^2+^, CTAC, and Br^−^ can generate significant color variations.

It has been reported that H_2_O_2_ can be hydrolyzed and therefore generates some powerful oxidant which can efficiently etch bulk Au^27^,^28^,^29^. However, the TMB^2+^ solution used in this work was produced by HRP-H_2_O_2_-TMB system, namely, the TMB^2+^ solution may contain H_2_O_2_ which may influence the process of TMB^2+^ etching. In this section, another comparative study was conducted to demonstrate whether H_2_O_2_ could influence the etching effect of TMB^2+^. As presented in Fig. 2A, compared with the blank (AuNPs with water), the mixture of H_2_O_2_, CTAB, and AuNPs shows seldom change in color and absorption value (530 nm). The absorption variations of AuNPs with different reaction time were compared between TMB^2+^ and H_2_O_2_ (Fig. 2B). The result shows that the etching time for TMB^2+^ and H_2_O_2_ was almost the same (within 1 min), that is, the residual H_2_O_2_ in TMB^2+^ solution has no influence on TMB^2+^ etching process. Many studies have reported the etching mechanism by various oxidizing agents in the past years. It has reported that the potential of AuBr_2_^−^-CTA^+^/Au is no more than 0.2 V (versus normal hydrogen electrode, NHE)^30^. Therefore, in the presence of CTA^+^ and Br^−^, many reagents can induce the etching of AuNPs. Zhang *et al*.^31^ reported that copper (II) can effectively etch gold nanorods in the presence of CTAB. Zhu *et al*.^32^ demonstrated that bromine anions were oxidized by hydrogen peroxide to form tribromide, which would then etch gold nanorods to Au^+^. Nevertheless, TMB^2+^ may be a stronger oxidant than hydrogen peroxide and can efficiently etch AuNPs in the presence of CTAB in this work.

The reaction process of TMB^2+^ and AuNPs was also studied by UV-vis spectrum. The maximum absorption peak of TMB is at 285 nm^6^. Figure 1C presents that the chromogenic substrate (TMB + H_2_O_2_) and TMB^2+^ solution both exhibited the characteristic absorption peak, and the absorption intensity of pure substrate was nearly three times than that of TMB^2+^. When TMB^2+^ mixed with AuNPs, the absorption intensity at 285 nm rose, but the absorption intensity at 450 nm decreased. This phenomenon suggests the TMB generated after mixing TMB^2+^ with AuNPs. The reaction relationship may follow the equation (1) shown below:


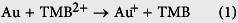


**Figure 3:**
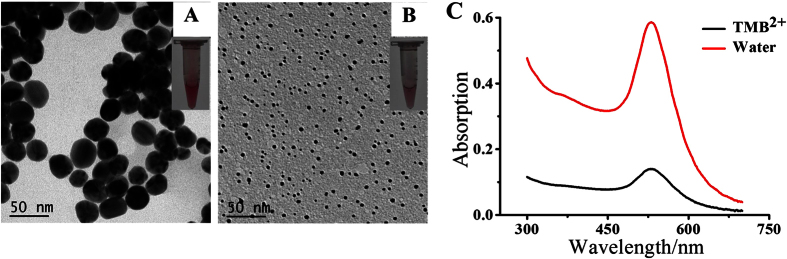
TMB^2+^ induced etching of AuNPs. (**A**,**B**) are TEM images of 50 μL AuNPs (~10 nM) mixed with 200 μL H_2_O and 200 μL 0.2155 mM TMB^2+^. The insets in (**A**,**B**) are corresponding images of the AuNPs solutions. (**C**) is the UV-vis absorption spectra corresponding to sample (**A**,**B**).

Figure 3A,B display the transmission electron microscopy (TEM) images of CTAB-capped AuNPs in the presence and absence of TMB^2+^. In the absence of TMB^2+^, the initial size of CATB-capped AuNPs is ~45 nm, and they are quasi-spherical; the solution color is deep wine red (Fig. 3A). After the addition of TMB^2+^, the size is ~4 nm (Fig. 3B). Figure 3C displays their corresponding UV-vis spectra, in which a significant decrease was observed at ~530 nm in the presence of TMB^2+^. These results also indicate that TMB^2+^ can effectively etch AuNPs.

### Optimization of experimental conditions

**Figure 4:**
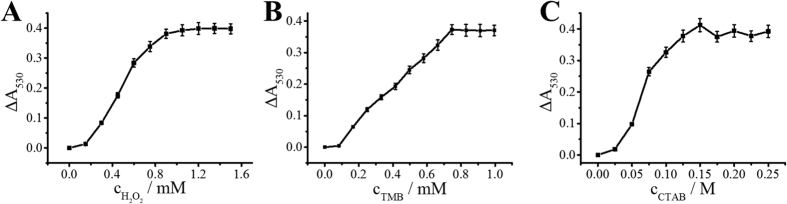
Effect of (**A**) H_2_O_2_ concentration, (**B**) TMB concentration and (**C**) CTAB concentration for TMB^2+^ etching AuNPs. Error bars represent standard deviations of three replicates.

To obtain the best etching effect, we investigated some experimental conditions. The colorimetric assay is based on the reaction between TMB^2+^ and AuNPs, so the production of TMB^2+^ is built on HRP-H_2_O_2_-TMB system. Hence, the concentrations of H_2_O_2_ and TMB were firstly evaluated. As shown in Fig. 4A,B, Δλ_530_ increased as the H_2_O_2_/TMB concentration increased and tended to stable at 1.2 mM (H_2_O_2_) and 0.75 mM (TMB). Thus the optimum concentrations of H_2_O_2_ and TMB were 1.2 mM and 0.75 mM, respectively. The CTAB concentration is another key factor for TMB^2+^ etching process. Figure 4C shows Δλ_530_ increased with the increment of CTAB concentration and reached a platform at 0.15 M. Therefore, 0.15 M CTAB was used for the following study.

### HRP concentration dependent etching of AuNPs

**Figure 5:**
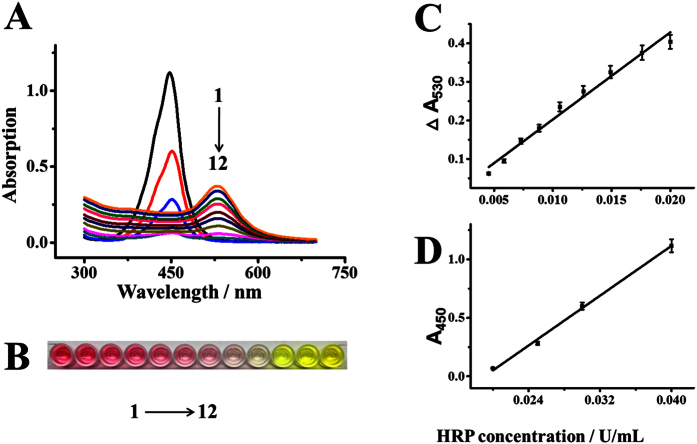
The UV-vis absorption spectra (**A**) images (**B**) and calibration curves (**C**,**D**) of the proposed dual-color ELISA for the detection of HRP. Concentrations of HRP for sample number 1 to 12 are 4.5, 5.8, 7.3, 8.8, 10.6, 12.6, 14.9, 17.6, 20, 25, 30, and 40 mU/mL, respectively. The calibration curves are divided into two sections: the wine red solutions are monitored by the changes of absorption value at 530 nm (ΔA_530_), and the yellow solutions are monitored by the changes of absorption value at 450 nm (A_450_). Error bars represent standard deviations of three replicates.

HRP is used as a label to indicate the presence of analyte in conventional ELISA, the concentration of analyte is therefore proportional to the concentration of HRP adsorbed on the substrate via antibody-antigen interaction. Hence firstly we assessed the relationship between the concentration of HRP and the color display of the solution. Figure 5A shows the UV-vis spectra of the system in response to different concentrations of HRP. The UV-vis spectrum of AuNPs initially exhibits a peak at ~530 nm. Upon the addition of HRP, the intensity significantly decreased. When the HRP concentration was 20 mU/mL, no obvious absorption peak was observed in the visible region (e.g. 400–800 nm) and the solution was colorless. The result indicates that no AuNPs exists in the solution. Figure 5B correspondingly displays the color variations from 1 to 12, and the intensity change at 530 nm (ΔA_530_) increased linearly with the HRP concentration ranging from 4 to 20 mU/mL (Fig. 5C). After the solution turned into colorless, further increase of HRP concentration made the solution turn into yellow. This yellow solution indicates the presence of access TMB^2+^. In this case, the optical density at 450 nm was used for the quantification of HRP (Fig. 5D), which is similar to conventional ELISA.

### Visual detection of PSA in human serum

**Figure 6:**
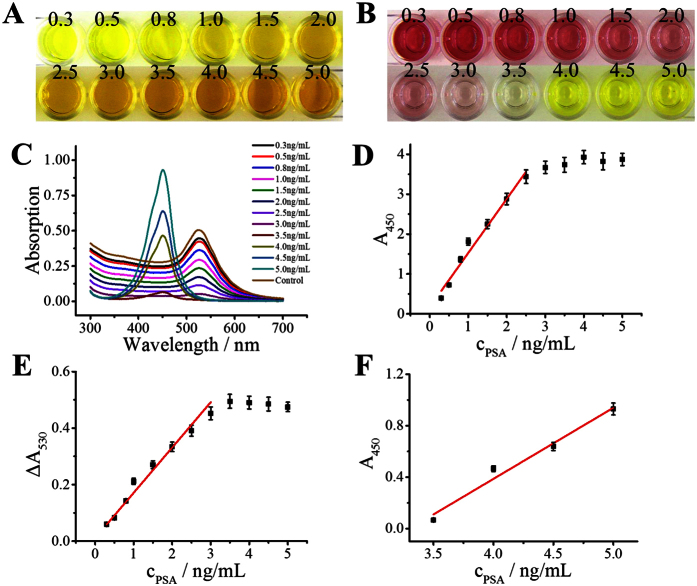
Compare between traditional ELISA and this dual-color ELISA. (**A**,**B**) were the photos of traditional ELISA and this dual-color ELISA in detection of PSA; (**C**) UV-vis spectrum of this dual-color ELISA; (**D**) calibration curve of the traditional ELISA; (**E**,**F**) were the calibration curve of wine red section and yellow section which monitored separately by the changes of absorption value at 530 nm (ΔA_530_) and absorption value at 450 nm (A_450_), respectively. Error bars represent standard deviations of three replicates.

The feasibility of the proposed method was evaluated for visual detection of PSA in human serum. A commercially available human PSA ELISA kit was adopted for the detection of PSA. All operation procedures were followed by the manual of the kit with the only difference in the color display step. The conventional ELISA was added a stop solution to turn the TMB^+^ into yellow TMB^2+^ (Fig. 6A), while we added an additional AuNPs solution into the yellow solution to display the dual colors (Fig. 6B). In the absence of PSA, the solution color was deep wine red, and the solution showed an absorption peak at ~530 nm. After the addition of PSA, the solution colors varied from wine red to colorless in the concentration range from 0.3 to 3 ng/mL; and then it displayed yellow when the concentration was more than 3 ng/mL (Fig. 6C). Compared with the conventional ELISA, this dual-color method shows at least two advantages. First, the conventional ELISA only shows one color (yellow) while our method shows red and yellow in response with different concentrations of PSA. This vivid color display could greatly improve the accuracy of visual inspection. Second, for conventional ELISA, the absorption value of 450 nm exceeded the acceptable range of microplate reader when the PSA concentration was more than 2.5 ng/mL (Fig. 6D); whereas the absorption value significantly decreased when AuNPs were added and then was signaled by the microplate reader. By record the decrease of absorption intensity at the wavelength of ~530 nm or absorption intensity increase at the wavelength of ~450 nm, two working curve which corresponding to the red region or yellow region were obtained (Fig. E,F). Other than conventional ELISA, this dual-color ELISA could detect PSA above 2.5 ng/mL, owing to the reason that part of the TMB^2+^ was consumed by AuNPs. These results indicate that this dual-color ELISA has a wider detection dynamic range than traditional ELISA. The limit of detection was 0.0093 ng/mL (3σ/slope), which is similar to traditional ELISA.

## Conclusion

In summary, we have developed a dual-color plasmonic ELISA for the sensitive detection of disease biomarkers with the naked eyes. Our investigation reveals that TMB^2+^ can quantitatively etch AuNPs, thus the addition of AuNPs to the solution of conventional HRP-TMB based ELISA can turn the single color (e.g. yellow) into dual color (e.g. red and yellow) display. This vivid color display improves the accuracy of visual inspection with the naked eyes. The dual-color plasmonic ELISA might have a potential promising market for the detection of many disease biomarkers that are presently detected with the conventional ELISA.

## Methods

### Reagents and materials

Gold chloride solution (HAuCl_4_) and TMB liquid substrate system were purchased from Aladdin (Shanghai, China). The sodium citrate, ascorbic acid, and sodium bromide were obtained from Fu Chen Chemistry (Tianjin, China), and CTAB was purchased from J & K Chemical Technology (Beijing, China). HRP and human total PSA ELISA kit were purchased from Sigma-Aldrich (USA). All other reagents were used as received. UV-vis absorption spectra were recorded with Tianmei UV 2310II (Shanghai, China) or Multiskan spectrum microplate spectrophotometer (Thermo, USA). Ultrapure water was from Direct-Q3 UV system (Millipore, 18.2 MΩ·cm), and all photos were taken by Canon EOS 600D digital camera.

### Preparation of the CTAB capped AuNPs

Au seeds were prepared according to previous method^33^. Briefly, 100 mL of HAuCl_4_ aqueous solution (2.5 × 10^−4^ M) was refluxed in the oil bath at 120 °C with vigorous stirring for 30 min, followed by adding 10 mL of sodium citrate (1%). The mixture solution was continued refluxing for 20 min. Then the gold seed solution was cooled down to the room temperature. CTAB-capped AuNPs were synthesized with a seed mediated method reported previously^34^. Briefly, CTAB (0.21862 g) was dissolved in HAuCl_4_ (60 mL, 2.5 × 10^−4^ M) and the mixture solution was incubated in water bath (30 °C). Then 3 mL of 0.1 M freshly prepared ascorbic acid was added followed by gentle stirring for 2 min. The color of the liquid turned into colorless when the ascorbic acid was added. Finally, 30 mL of Au seed solution was added and the mixtures were kept at 30 °C in a water bath for at least 6 h. The absorption peak of CTAB-capped AuNPs was ~530 nm, and the average diameter was ~45 nm calculated by the UV-vis adsorption^35^ (the detail can be seen in supporting information 2).

### Preparation of TMB^2+^ stock solution

The TMB^2+^ stock solution was prepared by HRP-H_2_O_2_-TMB system. Firstly, 100 μL of HRP (0.15 U/mL) was added to a test tube, followed by adding H_2_O_2_ (1 mL) and TMB (1 mL). The mixture turned into light blue quickly and then blue color deepened as time extended. When the enzymatic reaction was about 10 min, HCl (1 mL, 2 M) was added to the mixture to end the reaction. The color changed to yellow immediately, indicating that the TMB^2+^ is produced. According to previous literature^6^, the extinction coefficient of the TMB^2+^ is 5.9 × 10^4^ M^−1^ cm^−1^.

### Visual detection of HRP

To verify the effectiveness of this method, we investigated the relationship between HRP concentration and the color. Different concentrations of TMB^2+^ (150 μL) mixed with 100 μL of CTAB-capped AuNPs (~4.182 nM, the concentration was calculated by the UV-vis adsorption^36^, and the detail can be seen in supporting information 1). The color turned from red to colorless and then yellow, indicating the concentration of HRP is from low to high. Then we diluted HRP stock solution to different concentration solution. HRP (20 μL) mixed with H_2_O_2_ (100 μL) and TMB (100 μL), followed by adding HCl (80 μL, 2 M) after 10 min. The absorption value was measured at 450 nm.

### Detection of PSA in human serum

A human total PSA ELISA kit was adopted for the detection of PSA. Firstly, different concentrations of human PSA standard solution (100 μL) were added to well, followed by incubation for 2.5 h at room temperature. Aspirate and wash the wells three times with wash buffer. Then, 100 μL of biotinylated detection antibody was added to each well, followed by incubation for 1 h at room temperature with gentle shaking. After that, add 100 μL of HRP-Streptavidin conjugate to each well and incubate 45 min at room temperature with gentle shaking. Aspirate and wash the wells three times with wash buffer. Next, 100 μL of ELISA colorimetric TMB reagent was added to each well and incubate for 30 min at room temperature with gentle shaking. Then 50 μL of stop solution was added. Finally, 100 μL of AuNPs was added to each well and incubated for 5 min with gentle shaking. The absorption intensity was obtained with a microplate reader, and the photos were took in photo studio.

## Additional Information

**How to cite this article**: Guo, L. *et al*. Dual-color plasmonic enzyme-linked immunosorbent assay based on enzyme-mediated etching of Au nanoparticles. *Sci. Rep*. **6**, 32755; doi: 10.1038/srep32755 (2016).

## Supplementary Material

Supplementary Information
