# Supplementary material for: Dual-color plasmonic enzyme-linked immunosorbent assay based on enzyme-mediated etching of Au nanoparticles
Source: Sci Rep. 2016 Sep 7;6:32755. doi: 10.1038/srep32755 (PMC5013390; doi:10.1038/srep32755)
Supplement: Supplementary Information [file srep32755-s1.pdf]

Supporting Information:

Dual-color plasmonic enzyme-linked immunosorbent assay based on enzyme-mediated etching of Au nanoparticles

Longhua Guo, Shaohua Xu, Xiaoming Ma, Bin Qiu, Zhenyu Lin and Guonan Chen*

Institute of Nanomedicine and Nanobiosensing; Ministry of Education Key Laboratory of Analysis and Detection Technology for Food Safety; College of Chemistry, Fuzhou University, Fuzhou, 350116, China. Fax: (+) 86-591-22866135

E-mail: guolh@fzu.edu.cn

1. Calculate the concentration of AuNPs

The concentration of AuNPs was calculated by the previous study[1] according to:

$$C = \frac{A_{450}}{l \times \varepsilon_{450}}$$

where C represent the concentration of AuNPs; A_{450} is the absorption value at 450 nm; l is the optical path of gold sol, and ε_{450} is molar absorption coefficient of AuNPs at 450 nm.

2. Calculate the size of AuNPs

The method of calculate size of AuNPs has been reported early[2], the equation is following below:

$$d = \begin{cases} 3 + 7.5 \times 10^{-5} X^4, X < 23 \\ \dfrac{\left[\sqrt{X - 17} - 1\right]}{0.06}, X \geq 23 \end{cases}$$

where d is the average diameter of AuNPs, and X is datum which can got by the maximum absorption wavelength (λ_{max}) minus 500 ($X = \lambda_{max} - 500$).

References:

1 Haiss, W., Thanh, N. T., Aveyard, J. & Fernig, D. G. Determination of size and concentration of gold nanoparticles from UV-vis spectra. *Anal. Chem.* **79**, 4215-4221 (2007).

2 Khlebtsov, N. G. Determination of size and concentration of gold nanoparticles from extinction spectra. *Anal. Chem.* **80**, 6620-6625, doi:10.1021/ac800834n (2008).
